# How servant leadership motivates young university teachers’ workplace well-being: The role of occupational commitment and risk perception

**DOI:** 10.3389/fpsyg.2022.996497

**Published:** 2022-10-06

**Authors:** Jianji Zeng, Jiahui Lai, Xiaofan Liu

**Affiliations:** ^1^School of Medical Business, Guangdong Pharmaceutical University, Guangzhou, China; ^2^School of Pharmacy, Guangdong Pharmaceutical University, Guangzhou, China

**Keywords:** servant leadership, workplace well-being, occupational commitment, risk perception, young university teachers

## Abstract

Drawing on the integration of social exchange theory and situational power theory, this paper explores the effect of servant leadership on young university teachers’ workplace well-being and explores the mediating effect of occupational commitment and the moderating effect of risk perception on the indirect effects of servant leadership on workplace well-being. A questionnaire was distributed using the Questionnaire Star online questionnaire platform and a two-wave time-lagged design was used to collect 215 survey samples of young teachers from Chinese higher education institutions. SPSS 23.0 was used to test the hypothesized relationship between the variables. Results revealed that servant leadership was positively related to young university teachers’ workplace well-being. Occupational commitment plays a partial mediating role in linking servant leadership and young university teachers’ workplace well-being. Risk perception plays a moderating role in the indirect relationship between servant leadership, occupational commitment, and workplace well-being. When risk perception has a low level, the mediating effect of occupational commitment is stronger.

## Introduction

Workplace well-being is a form of well-being, which is an individual’s positive evaluation and emotional experience of the current work (Sun et al., 2016). In recent years, employees’ workplace well-being has been increasingly emphasized as the basis of organizational performance (Choi et al., 2017), because it can bring positive outcomes to employees and organizations (Wright and Cropanzano, 2004), such as job performance (Russell and Ea, 2008; Wang, 2015), engagement (Rasool et al., 2021), knowledge sharing, and individual innovation behavior (Wang et al., 2017; Liu et al., 2020). However, in the field of higher education in China, due to the central and local government vigorously constructing “double first-class” and “high level” universities, teachers, especially the young teachers, as the main undertaker and backbone of teaching and scientific research tasks in colleges and universities, are burdened with heavy workloads. Thus, they are subject to tremendous work stress and job burnout (Teles et al., 2020). Compared with other occupations, their workplace well-being is significantly lower (Grenville-Cleave and Boniwell, 2012). Young teachers with higher workplace well-being are more willing to invest time and energy in teaching and scientific research and are more willing to stay in school, help students grow and shape their values and belief systems (Yang et al., 2021; Ran et al., 2022), which may promote the development of universities (Wang, 2015). Therefore, it is of great significance to explore ways to improve young university teachers’ workplace well-being.

The existing literature shows that leadership, such as humble leadership (Zhong et al., 2020), inclusive leadership (Choi et al., 2017), and ethical leadership (Chughtai et al., 2015), is a key predictor of employee workplace well-being. However, incredibly, as far as we know, not many effects of servant leadership on employees’ workplace well-being have been specifically studied, especially in the field of higher education (Turner, 2022). Scholars have focused on task performance, employee creativity, knowledge hoarding, and other outcome variables of servant leadership (Chen et al., 2022; Zada et al., 2022a,b). Existing studies show that positive leadership behaviors can play a vital role in improving employees’ workplace well-being (Nielsen et al., 2008).Servant leadership, as a positive leadership, is honest and upright, selfless, and cares about helping subordinates develop (Eva et al., 2019). This people-oriented attitude helps to establish a harmonious relationship between leaders and subordinates, and creates an encouraging atmosphere for subordinates to realize their potential. This undoubtedly improves the possibility of subordinates’ workplace well-being (Ozturk et al., 2021). By exploring the influence of servant leadership on young university teachers’ workplace well-being, this study can contribute to servant leadership theory and empirical research. It also responds to Roberts (2020) call to conduct servant leadership research diverse settings, geographically, culturally, employment sector (private, government, and non-profit), and by type of occupation (Roberts, 2020).

In addition, the literature has explored servant leadership and occupational commitment (Long et al., 2014; Elsaied, 2021), and the relationship between occupational commitment and workplace well-being (Xu et al., 2021). However, in the relationship between servant leadership and workplace well-being, the mediating role of occupational commitment is rarely studied, which cannot well explain how servant leadership affects employees’ workplace well-being. Moreover, previous studies have shown that leadership style can affect employees’ workplace well-being through various mechanisms, calling for further research on different mediating variables in order to deeply understand the relationship between leadership style and employees’ workplace well-being (Chughtai et al., 2015; Rahimnia and Sharifirad, 2015). According to the social exchange theory, the employee-organization social exchange relationship is a non-contractual relationship of mutual benefit (Zeng and Ou, 2016). Due to the moral behavior of servant leaders and the priority of subordinates’ interests (Ehrhart, 2004), the uncertainty and risk in the employee-organization exchange relationship can be reduced. Occupational commitment is an important characteristic affecting employees and an important source of occupational meaning and continuity (Zhu et al., 2021). It influences employees’ response to the working environment (Valeau et al., 2019), which, in turn, affects employees’ work attitude or workplace well-being. Therefore, in order to make up for the lack of theoretical research and respond to the call of scholars, based on social exchange theory, this study explores the mediating effect of occupational commitment on the relationship between servant leadership and workplace well-being of young university teachers.

However, the research results of Zhong et al. (2020) show that the formation process of employees’ workplace well-being is not only influenced by positive leadership, but also moderated by situational factors. Moreover, leadership behavior is not always effective, and it may need supportive environment to function (Owens and Hekman, 2012). Therefore, from the perspective of servant leadership, we further investigated the effect of risk perception as a contextual variable on the effect of occupational commitment on workplace well-being of young university teachers. As an individual characteristic variable, risk perception is subjective and can reflect the degree to which an individual identifies a certain risk (Afolabi et al., 2021). Previous studies have shown that individual characteristics are important variables affecting employees’ workplace well-being (Siu et al., 2015). According to the situational power theory, risk perception, as a situational power, can provide important external cues for individual specific behavioral intentions (workplace well-being), and the strong situation (or weak situation) it creates will significantly hinder (or promote) the formation process of individual specific behavioral intentions (workplace well-being) (Meyer et al., 2010). Compared with high-risk perception, low-risk perception can enhance the workplace well-being of young teachers influenced by servant leadership through occupational commitment. Therefore, based on the situational power theory, it is helpful for us to further understand the formation mechanism of workplace well-being by identifying the boundary conditions of servant leadership affecting the workplace well-being of young university teachers.

The contribution of this study involves the following aspects. First, it explores the influence of servant leadership on young university teachers’ workplace well-being. Few previous studies have explored young teachers’ workplace well-being as an outcome variable of servant leadership (Eva et al., 2019). The conclusions of this study extend the effectiveness of servant leadership and at the same time enrich the antecedent variables of workplace well-being. Second, this study takes the psychological state of employees as the starting point and takes occupational commitment as an intermediary variable in the relationship between servant leadership and young teachers’ workplace well-being. The results, to a certain extent, reveal the black box of servant leadership affecting workplace well-being. Third, according to situational power theory, this study introduces the moderating variable of risk perception. Theoretically, it explains when and why servant leadership affects workplace well-being through occupational commitment (Meyer et al., 2010). The results show that high-risk perception can weaken the positive effect of occupational commitment and reduce workplace well-being, which enriches the contextual discussion of risk perception. Finally, the existing research objects of workplace well-being are basically limited to enterprise employees, and few scholars pay attention to and analyze the workplace well-being of a specific industry or group, especially the workplace well-being of young university teachers. It also enriches the study of workplace well-being.

## Theoretical background and hypotheses

### Servant leadership and workplace well-being

The term servant leadership was first proposed by Greenleaf, an American management scientist, in his book “The Servant Leader” published in 1970. He believed that a leader is primarily a servant rather than a leader. With the awareness of active service for employees, servant leaders try to meet the needs of employees, gain their trust of, and form the leadership that influences followers (Greenleaf, 1977). Servant leaders integrate servant and leadership. Leaders put their followers’ individual interests and needs above their own and are willing to empower employees by helping them grow and develop. The characteristics of servant leadership were put forward by Dennis and Bocarnea (2005), including humility, trust, empowerment, vision, and love for subordinates. Servant leadership can bring a series of positive results to employees, such as organizational citizenship behavior, employee engagement, performance, and so on (Liden et al., 2014; Panaccio et al., 2015; Canavesi and Minelli, 2021).

Well-being has been the goal pursued by people since ancient times. Well-being is an experience, an attitude, a personality characteristic, and a realm (Sun et al., 2016). Workplace well-being is derived from the study of well-being, which is considered to be an individual’s subjective positive experience at work (Chen et al., 2013). It consists of five aspects: interpersonal fit at work, thriving at work, feeling competent at work, perceived recognition at work, and desire for involvement at work (Dagenais-Desmarais and Savoie, 2012). It is of great significance to improve employees’ workplace well-being because workplace well-being is critical to the survival and development of any organization in the world (Spreitzer and Porath, 2012). Workplace well-being is considered to be the glue to retain and motivate high-quality employees, especially in an environment where the relationship between employees and organizations is loose (Fisher, 2010).

Servant leaders transcend personal interests and give priority to employees’ individual interests. They are not motivated by power but by serving others (Luthans and Avolio, 2003). Drawing on social exchange theory, when the leader, as an organizational agent, is willing to pay the cost of support and help to the employee, the employee will have a positive attitude and behavior to give back to the organization after receiving such help (Little et al., 2016). Thus, servant leadership can enhance employees’ workplace well-being. First, servant leaders have the virtue of humility, which reflects the servant leaders’ correct understanding of themselves and shows that servant leaders respect employees and recognize their contributions to the organization, which will make employees feel trusted and supported by leaders. Second, servant leaders are willing to empower employees. Employees are encouraged to make self-decisions and share information and innovation (Konczak et al., 2000). The purpose of a servant leader is to cultivate employees’ active and confident working attitudes, which indicates that servant leaders value employees and help them grow (Laub, 1999). Finally, servant leadership pays attention to the wishes and needs of employees, which is a remarkable feature, and it is different from other types of leaders. In contrast to transformational leadership, which focuses on the achievement of organizational goals, servant leadership focuses on the aspirations and goals of employees (Van Dierendonck, 2011). Previous studies have shown that leadership behaviors affect employees’ workplace well-being (Van Dierendonck et al., 2004; Wang et al., 2022). Thus, we hypothesize the following:

*Hypothesis 1 (H1)*: Servant leadership is positively related to young teachers’ workplace well-being.

### Mediating role of occupational commitment

Occupational commitment is an individual’s commitment to an occupation or a profession, which reflects the individual’s desire to stay in the current occupation and their degree of preference for the current occupation (Blau, 1985). Occupational commitment begins with individual learning and is reinforced throughout the occupational society (Chiang et al., 2016). It helps explain employee work behavior (Meyer and Herscovitch, 2001). Employees with high occupational commitment tend to invest more time and money to reach their occupation goals (Srikanth and Israel, 2012). Compared with teachers who have insufficient occupational commitment, teachers with occupational commitment perform better in their profession and organization, and thus help to improve the overall performance of the organization (Bogler and Somech, 2004). As a result, occupational commitment can improve employees’ workplace well-being, skill development, and occupation engagement, and leads to employees’ willingness to participate in occupation development programs (Vandenberghe and Ok, 2013).

Social support is an important variable affecting employees’ occupational commitment, and it has been empirically supported (Lin, 2020). Social support is a source-specific social variable that enables employees to better cope with the occupational environment they face. Sources of social support in the workplace include leaders, colleagues, and occupation counselors (Wolfgang, 1995). Servant leaders are a new leadership style that focuses on how leaders can help subordinates succeed, develop, and grow (Liden et al., 2014). Therefore, servant leaders are delegated to empower subordinates and encourage them to actively participate in their work, which strengthens their motivation to work in specific occupational roles. According to the reciprocity principle of social exchange theory, the behavior of leadership as an organizational agent will enhance the motivation of subordinates to work in a specific occupational role, and subordinates are willing to stay in the current occupation and invest more time and energy. Moreover, by encouraging the communication process and promoting the participation of subordinates in decision-making, servant leadership can create a pleasant organizational atmosphere and improve employees’ occupational commitment. This phenomenon is supported by the related literature, which shows that leadership plays an important role in employees’ occupational commitment (Long et al., 2014; Lin, 2020).

Occupational commitment reflects people’s motivation to work hard on personal development in their occupation. Employees with a high level of occupational commitment are more willing to invest time and money to participate in vocational training, skill development, and at work. They are more likely to get a better job performance. Based on the social exchange theory, an organization will give high-performance employees better salary, and more opportunities for promotion, so that they can derive more well-being from their work. To be specific, first, employees with high occupational commitment are more likely to identify with the value and purpose of their occupation. They can easily find the meaning and pleasure in their work, and are more likely to achieve career success (Fu, 2011). Career success can bring not only material satisfaction but also spiritual enjoyment, and can improve their workplace well-being. Second, employees with occupational commitment have more career satisfaction in their work, and satisfaction itself is a part of workplace well-being. Compared with employees with low occupational commitment, those with high occupational commitment have a higher level of workplace well-being. Thus, we hypothesize the following:

*Hypothesis 2 (H2)*: Occupational commitment mediates the servant leadership-workplace well-being relationship.

### The moderating role of perceived risks

In the organizational context, risks may be caused by factors internal to the work environment or external to the workplace (Alomari et al., 2018). If the probability of risk is higher and the consequences are more serious, people may think that the risk is greater. Risk perception is the degree to which an individual identifies a certain level of risk (Oppong, 2015). It is generally believed that individual risk perception mainly depends on intuition, emotion, and direct judgment (Slovic and Weber, 2002); thus, risk perception is subjective. Facing the same working environment, different people have different risk perceptions. Hence, what truly affects individual decision-making is not the actual risk but its perception. It is influenced by social, political, and psychological factors (Slovic, 1999).

Situational power theory points out that the formation of individual behavioral intention is affected by both the individual and the situation. External situational factors provide the individual with situational cues about individual behavior intention, which have an impact on the transformation process of individual cognition to specific behavior intention, either promoting or hindering (Meyer et al., 2010; García-Arroyo and Segovia, 2021). Studies have shown that work environment, leadership style, and individual perception are all common situational forces (Huang and Peng, 2015; Zhang et al., 2019). Young teachers perceive situational cues from the school about the fairness of career promotion and performance appraisal, which have an impact on the role of servant leadership on young teachers’ workplace well-being through their occupational commitment. As a result, risk perception may play a moderating role between occupational commitment and workplace well-being. Specifically, in the high-risk perception situation, young teachers receive more risks from the school in terms of career stability, career promotion fairness, and so on. This high-risk work environment has become a situational force, which weakens the positive impact of occupational commitment on workplace well-being. However, young teachers with low-risk perception can obtain more support and goodwill from the school and can enhance their trust in the school, thus enhancing the impact of occupational commitment on workplace well-being. Thus, we hypothesize the following:

*Hypothesis 3 (H3)*: Risk perception moderates the relationships between occupational commitment and workplace well-being in such a way that the relationships will be stronger for individuals with low-risk perception than those with high-risk perception.

Based on the above assumptions, we further propose a moderated mediation model; that is, different levels of risk perception moderate the indirect effect of servant leadership on workplace well-being through occupational commitment. Facing of the influence of occupational commitment, teachers with low-risk perception are more likely to identify with their occupation and devote themselves to their work, which leads to a higher sense of workplace well-being. In this case, the influence of servant leadership on young teachers’ workplace well-being is enhanced through occupational commitment. Instead, for teachers with high-risk perception, they will worry about the return of occupational investment, and the emotional dependence of occupation may also be reduced. According to the social exchange theory, young teachers’ workplace well-being tends to decrease, so the influence of servant leadership on young teachers’ workplace well-being through occupational commitment is weakened. To sum up, compared with high-risk perception, servant leadership has a greater impact on workplace well-being through occupational commitment in low-risk perception. Thus, we propose the following:

*Hypothesis 4 (H4)*: Risk perception moderates the indirect effect of servant leadership on young teachers’ workplace well-being via occupational commitment, such that the indirect effect is more positive with low rather than high risk perception.

**Figure 1:**
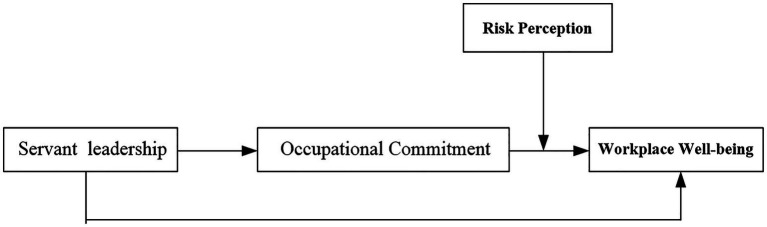
Theoretical model.

Based on the above arguments, we propose the following theoretical model (Figure 1).

## Materials and methods

### Participants and procedure

The survey samples for this study mainly come from young teachers in 20 universities in China, including major universities and ordinary universities. The “young teachers” in this study refer to full-time teachers under the age of 45 who are specialized in teaching and scientific research in institutions of higher learning. The data were collected using the Questionnaire Star (a professional online questionnaire platform) and emphasized the anonymity of the questionnaire and the purpose of academic research to eliminate the worries of the participants in filling out the questionnaire. To reduce the influence of homology deviation on the relationship between variables, the questionnaire was issued in two stages, with an interval of 2 weeks. Servant leadership and risk perception were assessed by a young teacher at Time 1. Workplace well-being and occupational commitment were measured by a young teacher at Time 2. Through the abovementioned questionnaire collection method and excluding unqualified questionnaires, such as incomplete and random filling of information, 215 valid questionnaires were finally obtained. Among the valid samples, in terms of gender, the proportion of women is slightly higher (111 female teachers, accounting for 51.6%; 104 male teachers, accounting for 48.4%). In terms of age, 26 teachers are under 30, accounting for 12.1%; 32 teachers are 31–35, accounting for 14.9%; 75 teachers are 36–40, accounting for 34.9%; and 82 teachers are 41–45, accounting for 38.1%. In terms of education level, 22 teachers have a bachelor’s degree or below, 107 have a master’s degree, and 86 have a doctoral degree, accounting for 10.2, 49.8, and 40%, respectively. In terms of professional titles, there are 117 lecturers or below, accounting for 54.4%; 85 associate professors, accounting for 39.5%; and 13 professors, accounting for 6%.

### Measures

To ensure the validity and reliability of the questionnaire, all measurement items were selected from established scales. On the basis of the pretest, some inappropriate expressions in the questionnaire were further revised. All variables were scored by a seven-point Likert scale.

#### Servant leadership

Servant leadership was measured by the scale revised by Sun and Wang (2010) based on the earlier work of Barbuto and Wheeler (2006), with a total of 15 items. A sample item is “my leader does everything he or she can to serve me.” The Cronbach’s α of the scale was 0.95.

#### Occupational commitment

Occupational commitment was measured by the scale developed by Blau (1989), with a total of 7 items. A sample item is “I love my profession very much and will not give up on it.” The Cronbach’s α was 0.89.

#### Risk perception

This construct was assessed using Huang (2009), with a total of 10 items. A sample item is “In school work, I encountered a bottleneck in the promotion of professional titles.” The Cronbach’s α of the scale was 0.79.

#### Workplace well-being

This construct was assessed using Zheng et al. (2015), with a total of 6 items. A sample item is “In general, I feel fairly satisfied with my present job.” The Cronbach’s α of the scale was 0.91.

## Results

### Common method variance

To avoid the common method deviation of the survey samples, the research group emphasized the anonymity and confidentiality of the information in the questionnaire, as well as the academic purpose of using the information in the questionnaire. In addition, this study adopted the test method recommended by the literature (Podsakoff et al., 2003) and used the Harman single factor method to perform EFA analysis on the research variables. The results showed that the first factor in the unrotated factor accounts for 32.72% of the variance, under the recommended value by 50%. Therefore, the common method deviation of the survey samples in this study was within the acceptable limits.

### Reliability and validity

This study adopted SPSS 23.0 statistical software to test the reliability and validity of the data. In terms of reliability, the analysis results of servant leadership, workplace well-being, occupational commitment, and risk perception showed that the Cronbach’s α coefficients of these four variables were 0.95, 0.89, 0.79, and 0.91, respectively, which were all greater than the usual standard of 0.7 and indicated that the collected data have good reliability.

In terms of validity, the data were tested for validity using principal component analysis and the maximum variance rotation method. The KMO values of the variables such as servant leadership, workplace well-being, occupational commitment, and risk perception were 0.92, 0.86, 0.80, and 0.87, respectively. The cumulative variance contribution rates were 76.099, 59.764, 59.268, and 69.018. The factor loadings of all items in the 4 scales were greater than 0.6. In summary, the data collected in this study have good reliability and validity.

### Descriptive statistics

The mean, standard deviation, and correlation coefficient of each variable are shown in Table 1. Table 1 shows that servant leadership, occupational commitment, and workplace well-being are significantly positively correlated; servant leadership was positively related to occupational commitment; and servant leadership, workplace well-being, and risk perception were negatively related. This laid the foundation for subsequent research.

**Table 1:** Correlation coefficients of each variable and Cronbach’s α.

Variables	Mean	SD	1	2	3	4
1. SL	3.86	1.26	(0.95)			
2. OC	5.09	1.06	0.18**	(0.89)		
3. PR	4.31	1.17	−0.27**	−0.01	(0.79)	
4. WWB	4.87	1	0.44**	0.50**	−0.17**	(0.91)

**p < 0.01. SL, servant leadership; WWB, workplace well-being; OC, occupational commitment; RP, risk perception.

### Hypothesis testing

This study used a hierarchical regression method to examine the effect of servant leadership on young teachers’ workplace well-being. First, demographic variables such as gender, age, education, and professional title were introduced into the regression model. Second, we put the servant leadership and control variables into the regression model (see Table 2). It could be seen from Model 1 that, except for gender, age, educational background, and professional title had no significant effect on young teachers’ workplace well-being. The research results showed that servant leadership had a significant positive effect on young teachers’ workplace well-being (β = 0.26, *p* < 0.01). Therefore, Hypothesis 1 was supported by the data.

**Table 2:** Results of hierarchical regression analysis.

Variables	Dependent variable: WWB						Mediator: OC	
	M_1_	M_2_	M_3_	M_4_	M_5_	M_6_	M_7_	M_8_	M_9_
Control variables									
Gender	−0.15*	−0.10	−0.17*	−0.12*	−0.09	−0.11*	−0.11*	0.05	0.05
Age	−0.00	0.05	−0.05	−0.00	0.06	0.00	−0.01	0.12*	0.12*
Education	−0.10	−0.04	−0.09	−0.04	−0.04	−0.03	−0.06	−0.00	−0.00
Title	−0.01	0.00	−0.04	−0.03	0.01	−0.02	−0.01	0.07	0.07
Research variables									
SL		0.43**		0.34**	0.42**	0.33**	0.32**	0.21**	0.21**
OC			0.51**	0.44**		0.44**	0.45**		
RP					−0.05	−0.05	−0.04		−0.01
OC × RP							−0.11*		
R^2^	0.32	0.21	0.29	0.39	0.21	0.39	0.41	0.06	0.06
⊿R^2^	0.13	0.19**	0.27**	0.38**	0.19**	0.37**	0.38**	0.03**	0.03**
F	1.71	11.09**	16.76**	22.39**	9.31**	19.26**	17.54**	2.48**	2.06**

**p < 0.01, *p < 0.05. SL, servant leadership; WWB, workplace well-being; OC, occupational commitment; RP, risk Perception.

In terms of the mediation effect test, the results in Model 2 met the first condition of the mediation effect test by Baron and Kenny (1986). Models 8, 3, and 4 met the second, third, and fourth conditions of the mediating effect test, respectively. The results of Model 8 (β = 0.21, *p* < 0.01) indicated that servant leadership had a significant impact on the occupational commitment of young teachers. In Model 3, occupational commitment had a significant impact on young teachers’ workplace well-being (β = 0.51, *p* < 0.01). Model 4 added occupational commitment as an intermediary variable. It can be seen from Model 4 that the coefficient of occupational commitment was significant (β = 0.44, *p* < 0.01), but the influence of servant leadership on workplace well-being was reduced, with still a significant positive impact (β = 0.34, *p* < 0.01). Therefore, occupational commitment had a partially mediating role between servant leadership and young teachers’ workplace well-being. Therefore, Hypothesis 2 was supported.

Regarding the moderating effect test, Muller et al. (2005) proposed the test method of moderating mediation. The first step was to examine the impact of independent variables and moderating variables on the dependent variable, with the regression coefficient of independent variables being significant; the second step was to examine the impact of independent variables and moderating variables on the mediating variables, with the regression coefficient of independent variables being significant; the third step was to examine the impact of independent variables, moderating variables and mediating variables on the dependent variable, with the coefficient of mediating variables being significant; and the fourth step was to examine the impact of independent variables, moderating variables, mediating variables, and interaction terms on dependent variables. The regression coefficient of the interaction terms should be significant in this fourth step. The meaning of each variable is the same as above; that is, the independent variable is servant leadership, the dependent variable is workplace well-being, the mediating variable is occupational commitment, the moderating variable is risk perception, and the interaction item is risk perception × occupational commitment.

The mediating role of occupational commitment had been verified. According to the above test steps, the regression coefficient of servant leadership of Model 5 in Table 2 was significant (β = 0.42, *p* < 0.01), and the regression coefficient of servant leadership in Model 9 was also significant (β = 0.21, *p* < 0.01). Model 6 showed that the regression coefficient of occupational commitment was significant (β = 0.44, *p* < 0.01), which again verified that the mediating role of occupational commitment was significant. Finally, Model 7 in Table 2 verified the impact of servant leadership, risk perception, occupational commitment, and interaction on workplace well-being. The regression coefficient of the interaction term was significant (β = 0.11, *p* < 0.05); that is, the moderating effect of risk perception was significant. These results indicated that both Hypotheses 3 and 4 were supported.

Bootstrapping analysis was used to further examine the moderated mediation effects. From the analysis results of the conditional indirect effects on the left part of Table 3, it could be seen that when young teachers’ risk perception level was low, the indirect effect of servant leaders on workplace well-being through occupational commitment was 0.08, and the confidence interval was [0.02, 0.14]. When the teacher’s risk perception level was high, the indirect effect of servant leaders on workplace well-being through occupational commitment was 0.05, and the confidence interval was [0.01, 0.11]. Since the above confidence interval did not contain a zero point, it meant that no matter whether the risk perception modifier took a low or high value, the indirect effect of service leaders on young teachers’ workplace well-being through occupational commitment was significant. The right half of Table 3 reported the relevant judgment index value INDEX obtained by SPSS Process calculation. That is, the judgment index of the indirect relationship between servant leadership and the workplace well-being for young teachers through occupational commitment was −0.01, the standard error was 0.01, and the confidence interval was [−0.0291, −0.0001]. Because the above confidence interval did not include the zero point, it indicated that the mediating effect of servant leadership on young teachers’ workplace well-being was significant. Hypotheses 3 and 4 were further supported.

**Table 3:** Bootstrapping analysis results of the moderated mediation.

Mediators	Indirect effect					Moderated mediation effect			
	Moderator	Effect	SE	95% LLCI	95% ULCI	INDEX	SE	95% LLCI	95% ULCI
OC	Low value	0.08	0.03	0.02	0.14	−0.01	0.01	−0.0291	−0.0001
	High value	0.05	0.02	0.01	0.11				

Resampling times = 5,000.

**Figure 2:**
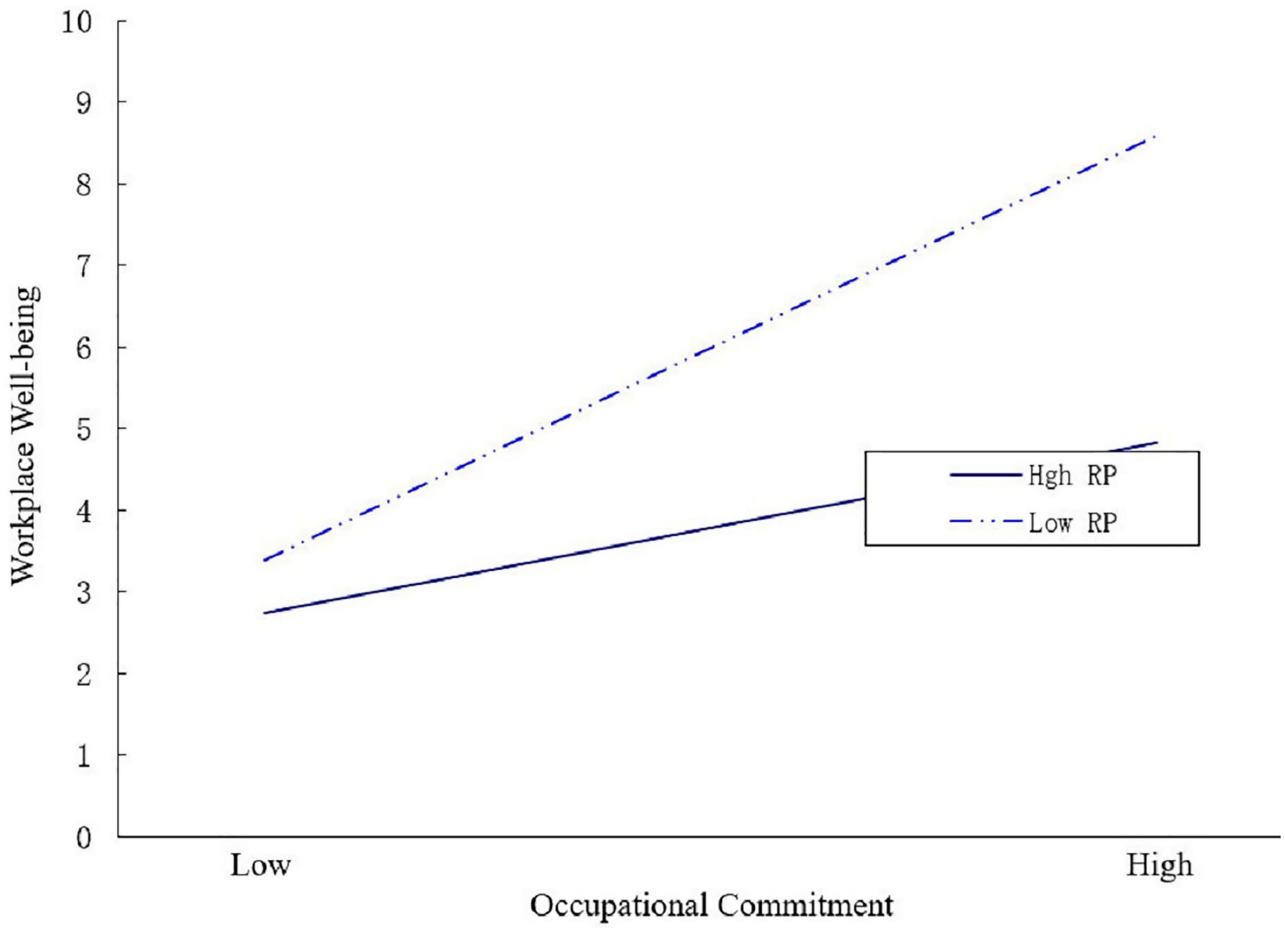
Interactive effects of occupational commitment and risk perception on workplace well-being.

To clearly describe the moderating effects of risk perception, we adopted the methods and procedures developed by Aiken and West (1991). The moderating effects of higher (*M* + *SD*) and lower (*M−SD*) risk perception on workplace well-being were plotted (see Figure 2). From Figure 2, it could be seen that low-risk perception could reinforce the positive impact of workplace well-being compared with high-risk perception.

## Discussion

### Theoretical implications

The theoretical contributions of this research mainly include the following:

Firstly, the research explains and verifies the effect of servant leadership in the field of higher education on the workplace well-being of young teachers. The findings suggest that this is consistent with many other studies, claiming that employee well-being can be maintained and improved through a certain leadership style (Chughtai et al., 2015; Zhong et al., 2020). This indicates that servant leadership is helpful to maintain and improve the workplace well-being of young teachers. Compared with previous studies, most of which focus on workplace well-being of employees in profit organizations (Miao and Cao, 2019; Fang et al., 2022), this study based on the research on workplace well-being of young university teachers, extends the research on workplace well-being in non-profit organizations to some extent.

Secondly, this study provides a new perspective for understanding the effect of servant leadership on subordinates’ workplace well-being. Although previous studies have paid attention to the importance of employee well-being in non-profit organizations such as higher education and preliminarily discussed it theoretically (Turner, 2022). However, this study not only empirically tested the relationship between servant leadership and young teachers’ workplace well-being, but also further explored the influencing mechanism of servant leadership on young teachers’ workplace well-being. The results show that servant leadership improves the well-being of young teachers at work by fostering their occupational commitment. In other words, young teachers under servant leadership can continuously enhance their occupational commitment and thus have a higher sense of workplace well-being.

Finally, this study reveals the boundary conditions of the influence of servant leadership on workplace well-being, and enriches the research on the situational factors of the influence mechanism of workplace well-being. A recent review of the research on workplace well-being shows that, in the related research on workplace well-being, scholars pay more attention to the antecedent and outcome variables of workplace well-being, but there are few studies on the boundary conditions of the formation mechanism of workplace well-being (Pang et al., 2018). Therefore, this study introduces risk perception into the influencing mechanism model of servant leadership on workplace well-being, and reveals that the effect of servant leadership on young teachers’ workplace well-being is subject to the moderating effect of young teachers’ risk perception, highlighting the contingency mechanism of servant leadership on young teachers’ workplace well-being.

### Practical implications

The significance of the research conclusions for practice is mainly manifested in the following:

First, school management should raise the awareness of leaders at all levels of the positive impact of servant leadership on young teachers’ workplace well-being. In actual work, it promotes a servant leadership style and strengthens practical training on servant leadership. In addition, schools should formulate human resource policies to promote the effectiveness of servant leadership. Second, schools should pay attention to the occupational commitment of young teachers. The school creates a good environment for young teachers in terms of on-the-job training, graduate tutor qualifications, etc., helps young teachers grow professionally, and enhances their occupational commitments. Finally, the moderating role of risk perception is of great significance for improving the effectiveness of servant leadership. In fact, the effectiveness of servant leadership is conditional. At work, given the school’s academic performance appraisal, title promotion, salary, and other matters concerning the core interests of young teachers, schools should have a clear system to ensure that young teachers have a stable expectation, reduce the risk perception of young teachers, and improve young teachers’ workplace well-being.

### Limitations and future research

This study also has some limitations: (1). To reduce the common method bias, although two time points were used to collect the research samples, the cross-sectional design made it difficult to avoid related effects. Future studies can use a longitudinal design to test the causal relationship. (2) The research sample only takes young university teachers as the object. The data source is relatively singular, and the universality of research conclusions may be insufficient. Future research can expand the sample to ordinary employees, especially healthcare workers (Zeb et al., 2021), so as to improve the universality of the research conclusions. (3) The effect size of the moderated mediation is relatively low, which may be related to the sample size. Future studies should appropriately expand the sample size to highlight the moderated mediation effect. (4) The research only examined the effect of the context variable of risk perception on workplace well-being through occupational commitment by service leaders. Future research can focus on the moderating effects of other contextual factors to enrich the extent of contextual variables.

## Conclusion

Based on the integration of social exchange theory and situational power theory, this study constructs and verifies the influence mechanism model of servant leadership on young teachers’ workplace well-being from the perspective of the Chinese cultural context, and obtains some research conclusions with theoretical and practical value. Specifically, we found that servant leadership has a significant impact on young teachers’ workplace well-being, and occupational commitment plays a mediating role between servant leadership and young teachers’ workplace well-being. Moreover, risk perception plays a moderating role in the indirect effect of servant leadership on workplace well-being through occupational commitment; that is, compared with young teachers with low-risk perception, this indirect effect is weakened under high-risk perception.

## Data availability statement

The raw data supporting the conclusions of this article will be made available by the authors, without undue reservation.

## Ethics statement

The studies involving human participants were reviewed and approved by the School of Medical Business at Guangdong Pharmaceutical University. The participants provided their written informed consent to participate in this study.

## Author contributions

JZ contributed to the conceptualization, methodology, software, validation, formal analysis, investigation, resources, data curation, and funding acquisition. JZ, JL, and XL contributed to the original draft preparation and writing—review and editing. All authors contributed to the article and approved the submitted version.

## Funding

This research was funded by Guangdong Planning Project of Philosophy and Social Science (GD21CJY02), Guangdong Planning Research Project of Education and Science (2020GXJK405), Guangdong University Project of Party Construction Research Association (2020BK145), and the Innovation and Strengthening University Project of Guangdong Pharmaceutical University (2018WTSCX053).

## Conflict of interest

The authors declare that the research was conducted in the absence of any commercial or financial relationships that could be construed as a potential conflict of interest.

## Publisher’s note

All claims expressed in this article are solely those of the authors and do not necessarily represent those of their affiliated organizations, or those of the publisher, the editors and the reviewers. Any product that may be evaluated in this article, or claim that may be made by its manufacturer, is not guaranteed or endorsed by the publisher.
